# Engineering Strategies for Allogeneic T Cell-Based Platforms in Cancer Immunotherapy

**DOI:** 10.3390/ph19070991

**Published:** 2026-06-25

**Authors:** Su-Jin Kang, Hyang-Mi Lee

**Affiliations:** College of Pharmacy, Dongduk Women’s University, Seoul 02748, Republic of Korea; sjkang@dongduk.ac.kr

**Keywords:** allogeneic T cell therapy, tumor immunotherapy, non-conventional T cells, multiplexed genome editing, cell therapy manufacturing

## Abstract

Allogeneic T cell therapies have emerged as a promising strategy to overcome the logistical and manufacturing limitations of autologous approaches, enabling scalable, off-the-shelf cancer immunotherapy. While early clinical efforts have focused predominantly on αβ T cell-based platforms, including CAR- and TCR-engineered approaches, a growing spectrum of alternative cell types, such as γδ T cells, invariant natural killer T cells, mucosal-associated invariant T cells, and induced pluripotent stem cell-derived effectors, is expanding the design landscape of allogeneic therapies. However, clinical translation remains constrained by immune rejection, limited persistence, lymphodepletion-associated toxicity, manufacturing variability, and impaired efficacy in solid tumors. To address these barriers, engineering strategies have increasingly integrated T cell receptor disruption, human leukocyte antigen modulation, cytokine support, checkpoint editing, and synthetic circuit design. This review provides an oncology-focused, cross-platform framework for evaluating diverse allogeneic T cell and T cell-like platforms according to clinical maturity, safety, manufacturability, persistence, and tumor-targeting capacity. We further discuss how platform-specific biological properties and clinical evidence can be integrated with modular engineering strategies to optimize antitumor performance. These insights support a shift from platform-centric development toward a design-driven paradigm for next-generation allogeneic cellular immunotherapies with improved efficacy, safety, and scalability.

## 1. Introduction

The clinical landscape of oncology has been fundamentally transformed by the success of adoptive cell therapy (ACT), particularly chimeric antigen receptor (CAR) T cell platforms. In the treatment of refractory hematological malignancies, CAR-T therapies have achieved unprecedented objective response rates (ORR), effectively converting previously incurable diseases into manageable clinical conditions [1,2,3,4]. These advances have established engineered T cells as a central modality in cancer immunotherapy and have catalyzed the broader development of cell-based therapeutic strategies for malignant disease [5,6].

Despite these successes, the autologous manufacturing paradigm, whereby a patient’s own T cells are harvested, genetically modified, and reinfused, poses significant logistical and biological limitations. High production costs and prolonged manufacturing timelines can delay treatment beyond the survival window of patients with aggressive disease. Moreover, prior therapies often compromise T cell fitness, leading to T cell exhaustion, reduced proliferative capacity, and occasional manufacturing failure [7,8,9]. These challenges contribute to variability in product quality and limit the scalability and accessibility of current ACT approaches.

**Figure 1:**
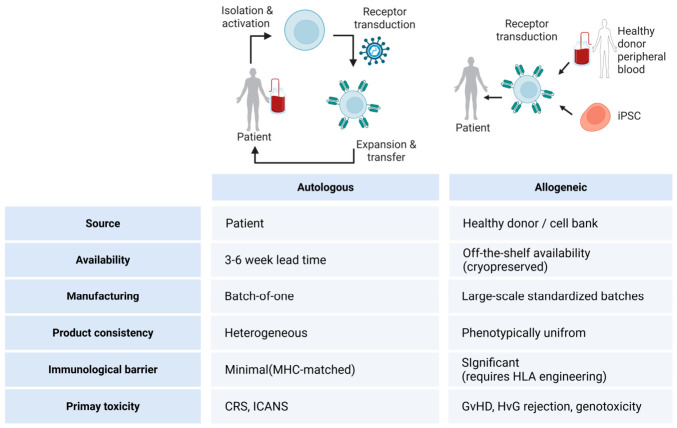
Autologous versus allogeneic T cell therapy platforms. Schematic representation of manufacturing workflows and key features of autologous and allogeneic T cell therapies. Autologous therapies (**left**) involve patient-derived T cells that are isolated, genetically modified, expanded, and reinfused as individualized products with inherent variability and longer production times. In contrast, allogeneic therapies (**right**) use cells from healthy donors or stem cell sources such as iPSC, enabling large-scale, standardized, cryopreserved “off-the-shelf” products. Key differences are highlighted across source, availability, manufacturing scalability, product consistency, immunological barriers, and associated toxicities.

To overcome these constraints, the field is increasingly shifting toward allogeneic, or “off-the-shelf,” T cell therapies. By utilizing cells derived from healthy donors or renewable sources such as induced pluripotent stem cells (iPSCs), allogeneic ACT enables the production of standardized, cryopreserved cell products that are readily available for immediate clinical use [7,10]. This approach has the potential to improve manufacturing efficiency, enhance product consistency, and expand patient access (Figure 1). Concurrently, the scope of effector cell platforms is broadening beyond conventional αβ T cells to include alternative subsets with intrinsic properties favorable for allogeneic application. For example, γδ T cells recognize antigens in a largely major histocompatibility complex (MHC)-independent manner, thereby reducing the risk of alloreactivity [11,12,13]. In addition, invariant natural killer T (iNKT) cells and mucosal-associated invariant T (MAIT) cells exhibit innate-like effector functions combined with features of adaptive immunity, including tissue homing and memory potential, which may enhance activity within the immunosuppressive tumor microenvironment (TME) [14,15,16]. These emerging platforms provide complementary biological properties that may help address key limitations of conventional αβ T cell therapies.

However, the successful clinical implementation of allogeneic ACT is fundamentally constrained by two major immunological barriers: graft-versus-host disease (GvHD) and host-versus-graft (HvG) rejection. GvHD arises from donor-derived αβ T cell recognition of recipient alloantigens, whereas HvG responses reflect immune-mediated clearance of infused cells by the host [17,18,19,20]. Together, these processes limit both the safety and persistence of allogeneic products and represent central challenges in their clinical translation. Advances in genome engineering and cell design have enabled the development of strategies to mitigate these risks while improving functional performance. These include approaches to modulate immune recognition, enhance persistence, and optimize cellular fitness. At the same time, increasing platform diversity and engineering complexity have introduced new considerations in manufacturing, regulatory evaluation, and clinical implementation.

Despite rapid progress, the clinical translation of allogeneic T cell therapies is constrained by fundamental immunological barriers, particularly GvHD and HvG rejection. These challenges, together with limited persistence and variable efficacy, highlight the need for integrated design strategies. In this review, we provide a framework that links immunological constraints with engineering solutions across diverse allogeneic platforms, including γδ T cells, iNKT cells, MAIT cells, and iPSC-derived effectors. We further compare these modalities and discuss engineering strategies to enhance persistence, immune evasion, and clinical efficacy.

To provide a comprehensive overview of the field, relevant literature was identified through searches of PubMed, Web of Science, Google Scholar, ClinicalTrials.gov, and the EU Clinical Trials Register for records published or available up to May 2026. Search terms included combinations of allogeneic or off-the-shelf cell therapy terms; platform terms such as CAR-T, TCR-T, γδ T cells, iNKT cells, MAIT cells, and iPSC-derived T cells; engineering terms such as TCR α constant (TRAC) knock-out, β2-microglobulin (B2M) knock-out, human leukocyte antigen (HLA)-E, HLA-G, CD52, and immune evasion; and clinical terms such as GvHD, HvG, persistence, hematologic malignancy, and solid tumor. Clinical studies were prioritized when supported by directly traceable primary sources, particularly trial registry records and peer-reviewed clinical reports. Review articles were used primarily for background context and cross-validation of platform development. Clinical entries discussed throughout this review were assessed in relation to the type and maturity of supporting evidence to facilitate transparent interpretation of the evolving clinical landscape.

## 2. Immunological Barriers in Allogeneic T Cell Therapies

The clinical development of allogeneic T cell therapies is fundamentally constrained by the immunological incompatibility between donor-derived cells and the recipient’s immune system. Unlike autologous approaches, allogeneic platforms must simultaneously prevent donor-mediated toxicity and resist host immune rejection. These dual challenges, GvHD and HvG responses, define the central design constraints for off-the-shelf T cell immunotherapy and have driven the emergence of specific engineering principles guiding next-generation platform development.

GvHD arises from the recognition of host alloantigens by donor-derived TCRs, driven by polymorphic differences in MHC molecules. This process leads to widespread immune activation and tissue damage, presenting a major safety risk in conventional αβ T cell-based platforms [19,20]. The severity of GvHD is influenced by both the degree of HLA mismatch and the functional state of infused T cells, making it a critical consideration in clinical application. As a result, uncontrolled TCR-mediated alloreactivity is a primary limitation for the use of donor-derived T cells. In parallel, HvG responses mediate the elimination of infused cells through both adaptive and innate immune mechanisms. Recipient T cells can recognize mismatched donor HLA molecules, while natural killer (NK) cells detect the absence of self-HLA class I expression through “missing-self” recognition [17,21]. These processes act in concert to limit engraftment and persistence, often leading to rapid clearance of allogeneic cells. The intensity of these responses can vary depending on the patient’s immune status and prior treatment, contributing to heterogeneous clinical outcomes and limiting the durability of therapeutic responses.

Beyond immune rejection, the efficacy of allogeneic T cell therapies is further restricted by intrinsic limitations in cellular persistence and functional fitness. Sustained antitumor activity requires that infused cells undergo robust expansion, maintain viability, and retain effector function over time. However, allogeneic cells are particularly susceptible to premature clearance and exhaustion due to continuous immune pressure and suboptimal cellular state [17,22]. These intrinsic limitations can significantly reduce the magnitude and duration of therapeutic responses. Extrinsic factors within the TME further compound these challenges, particularly in solid tumors. Immunosuppressive cytokines, hypoxia, and regulatory immune populations can impair T cell activation and effector function. In addition, inefficient trafficking and limited infiltration into tumor sites limit effective target engagement, further reducing therapeutic efficacy [16]. These extrinsic barriers act in concert with intrinsic cellular limitations, creating a complex and multifactorial landscape that constrains the performance of allogeneic T cell therapies.

## 3. Clinically Advanced Allogeneic αβ T Cell Therapies

The clinical translation of allogeneic T cell therapies has progressed rapidly over the past decades, with multiple platforms entering early-phase trials (Table 1). These efforts have primarily focused on αβ T cell-based CAR therapies, leveraging established targets from autologous settings while incorporating genome engineering strategies to mitigate alloreactivity. Initial clinical results support the feasibility of off-the-shelf approaches, although challenges in persistence and durability are central limitations.

### 3.1. First-in-Human Studies and Proof-of-Concept

The earliest clinical validation of allogeneic CAR-T therapy was demonstrated using UCART19, a transcription activator-like effector nuclease (TALEN)-engineered anti-CD19 CAR-T product derived from healthy donors. This platform incorporates disruption of the TRAC locus to eliminate endogenous TCR expression and CD52 knock-out to enable selective lymphodepletion. In a landmark study, UCART19 induced molecular remission in infants with relapsed or refractory B cell acute lymphoblastic leukemia, establishing proof-of-concept for universal CAR-T therapy [41]. Subsequent phase I trials further evaluated UCART19 in both pediatric and adult populations (e.g., NCT02746952, NCT02808442), presenting manageable safety profiles and evidence of antitumor activity [25,26]. These studies confirmed that genome-edited donor-derived T cells could be administered without inducing severe GvHD, thereby validating the central premise of TCR disruption as a safety mechanism. More recent clinical data further support the feasibility of allogeneic T cells across hematological malignancies [42].

In contrast, clinical experience with allogeneic TCR-engineered T cells is limited. Nonetheless, TCR-T therapies represent a fundamentally distinct targeting modality by enabling recognition of intracellular tumor antigens presented as peptide-HLA complexes. Evidence from autologous settings, particularly targeting NY-ESO-1, has shown robust antitumor activity in solid and hematological malignancies, establishing the therapeutic potential of this approach [43,44]. Translation into the allogeneic setting, however, is more complex due to HLA restriction and the risk of alloreactivity mediated by endogenous TCRs, necessitating extensive genome engineering [45].

### 3.2. Expansion of Allogeneic CAR-T Platforms

As shown in Table 1, early efforts have primarily centered on allogeneic CAR-T therapies in hematological malignancies, where these platforms have exhibited the most mature clinical activity. Clinical development is also extending into solid tumors and selected TCR-based approaches targeting intracellular antigens. This expansion reflects both technological advances in genome engineering and a growing understanding of tumor-specific immunological contexts.

In B cell malignancies, the phase 2 ALPHA3 trial, built on ALPHA (NCT03939026) and ALPHA2 (NCT04416984) studies, evaluates cemacabtagene ansegedleucel (cema-cel; ALLO-501), a TALEN-edited anti-CD19 product, as consolidation therapy for large B cell lymphoma (LBCL) patients who remain minimal residual disease-positive after standard R-CHOP [27,29,46]. Clinical data from ALPHA and ALPHA2 showed an ORR 67% and CR 58%, with early evidence of durability in CAR-T naïve LBCL, while the ongoing ALPHA3 trial is extending its use into MDR-positive settings [46]. Comparable efficacy appears in BALLI-01 (NCT04150497) with lasme-cel (UCART22) achieving ~60% CR in relapsed or refractory B-ALL using “Process 2” manufacturing [23,34,47], and UNIVERSAL trial (NCT04093596) of BCMA-targeted ALLO-715 showing 67% ORR in multiple myeloma [23].

This foundation enabled extension into solid tumors, where therapeutic resistance is driven by limited T cell infiltration and an immunosuppressive TME. Early clinical signals nevertheless indicate emerging activity. The TRAVERSE trial for ALLO-316 (CD70-targeted) reported 31% ORR in renal cell carcinoma with high CD70 expression (TPS ≥ 50%), indicating expansion into immunosuppressive “cold” TMEs [38,48]. Evidence for CLDN18.2-targeted CAR-T activity in gastric adenocarcinoma is derived primarily from autologous platforms and is therefore discussed only as contextual support for antigen selection rather than as direct evidence of allogeneic CAR-T efficacy [49]. Although efficacy is less consistent than in hematological malignancies, these studies mark a critical inflection point, suggesting that allogeneic CAR-T approaches can function within traditionally refractory tumor contexts.

Building on CAR-T momentum, the field has diversified into allogeneic TCR-T, which overcomes the antigenic limitations of CAR-based targeting by recognizing intracellular peptides presented on MHC molecules. Allogeneic TCR-T approaches are being adapted to address their unique biological constraints. HLA restriction is a defining limitation, typically requiring targeting of shared alleles such as HLA-A*02:01, thereby restricting patient eligibility. Genome editing to eliminate endogenous TCR expression is essential to reduce GvHD risk, while additional modifications may be required to mitigate HvG responses [44]. Enabled by precise genome editing strategies, including TRAC-targeted integration to eliminate endogenous TCR expression, these platforms extend the reach of allogeneic therapies to a substantially larger antigenic landscape [50]. In hematological malignancies, TSC-100 and TSC-101 (targeting MiHA HA-1 and HA-2, respectively) in the phase 1 ALLOHA study (NCT05473910) have achieved effective donor chimerism post-haploidentical HCT in AML/ALL/MDS without GvHD [40]. This paradigm further extends to solid tumors and chronic infections, where TCR-T therapies exploit their ability to recognize intracellular and viral antigens. Trials targeting NY-ESO-1, MAGE-A4, and PRAME are transitioning from autologous to allogeneic platforms using clustered regularly interspaced short palindromic repeats (CRISPR) or TALEN-mediated TRAC disruption, addressing patient variability and manufacturing delays. These outcomes confirm “off-the-shelf” feasibility while establishing engineering requirements for next-generation universal cell therapies [50]. More broadly, recent analyses of TCR-T clinical development have shown continued expansion of TCR-engineered therapies toward a wider range of intracellular tumor antigens and increasingly sophisticated gene-engineering strategies, highlighting the growing translational interest in TCR-based cellular immunotherapy [51].

These advances delineate a clear trajectory in which allogeneic CAR-T therapies are expanding beyond hematological malignancies into solid tumors, while TCR-T platforms further extend this reach to intracellular and infection-associated tumor targets. Despite these advances, limited persistence is a consistent challenge across allogeneic CAR-T studies. While initial responses are often achieved, long-term durability is frequently compromised, largely due to host immune rejection. These findings highlight the need for improved strategies to enhance in vivo activity. Importantly, these observations should be interpreted with caution because many products remain in dose-escalation or early expansion cohorts, and response rates often derive from small, heterogeneous populations with different lymphodepletion regimens and follow-up durations.

### 3.3. Safety, Resistance, and Strategies to Enhance Clinical Durability

Despite encouraging clinical activity, allogeneic CAR-T therapies are constrained by safety considerations and limited durability [25]. While severe GvHD is largely mitigated through TCR disruption, host-mediated rejection continues to restrict persistence and contributes to variability in clinical responses. In addition, lymphodepleting conditioning regimens, often required to facilitate engraftment, introduce risks such as prolonged cytopenias and increased susceptibility to infections [23,46,52]. For TCR-T therapies, safety risks extend beyond alloreactivity to include on-target, off-tumor toxicity and unintended cross-reactivity with structurally similar peptides, as highlighted in clinical studies of affinity-enhanced TCRs [53]. These risks underscore the importance of careful antigen selection and preclinical validation.

Beyond safety, resistance mechanisms further constrain therapeutic efficacy. CAR-T cells are primarily affected by antigen loss or modulation, whereas TCR-T cells depend on intact antigen processing and presentation pathways, making them susceptible to tumor escape through downregulation of HLA molecules. In the allogeneic setting, these intrinsic resistance mechanisms are compounded by host immune rejection, which limits durable engraftment across both platforms [7]. To address these limitations, current strategies focus on enhancing persistence and functional potency through integrated engineering approaches. These include modulation of HLA expression to evade immune recognition, incorporation of cytokine support systems such as interleukin-7 or interleukin-15 to promote survival and memory-like phenotypes, and optimization of receptor design to improve signaling and reduce exhaustion [22,54]. In parallel, modulation of inhibitory pathways such as programmed cell death protein 1 (PD-1) is being explored to sustain T cell function under chronic antigen exposure [55]. These strategies aim to improve persistence and sustain functional activity, which are key determinants of clinical durability in allogeneic T cell therapies.

Notably, although clinical development of allogeneic TCR-T cells is comparatively less advanced than that of CAR-T approaches, their distinct antigen recognition biology introduces unique opportunities and constraints, including HLA restriction and the risk of off-target reactivity. These features require tailored engineering strategies to achieve durable therapeutic responses [55]. Durable clinical responses will depend on coordinated optimization of immune evasion, cellular fitness, and functional stability, rather than reliance on any single intervention.

## 4. Alternative Allogeneic Platforms Beyond αβ T Cells

**Figure 2:**
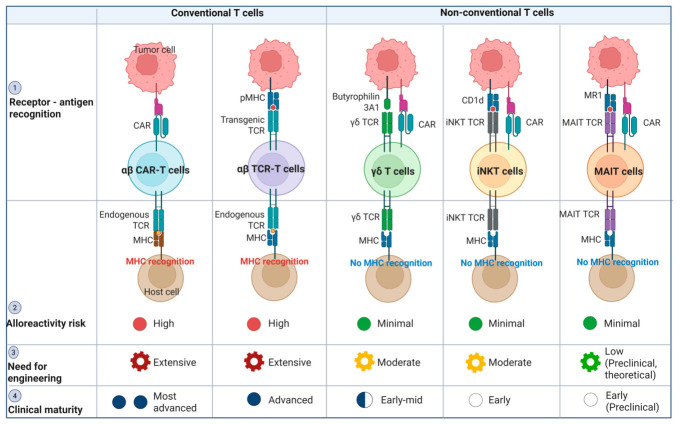
Functional and translational differences across allogeneic T cell platforms. Comparative summary of antigen-recognition mechanisms, alloreactivity risk, engineering requirements, and clinical maturity across major T cell platforms. Conventional αβ T cell platforms (CAR-T and TCR-T) rely on CAR-mediated surface antigen recognition or transgenic TCR-mediated peptide-MHC recognition, but retain a higher risk of host MHC recognition through endogenous or introduced αβ TCRs, requiring extensive genetic modification to reduce alloreactivity. In contrast, non-conventional platforms, including γδ T, iNKT, and MAIT cells, utilize MHC-independent or monomorphic antigen-presenting molecule-restricted recognition pathways, such as butyrophilin-associated, CD1d-restricted, or MR1-restricted recognition. These innate-like platforms generally show lower alloreactivity risk and reduced engineering requirements, although their clinical development remains less mature than that of αβ T cell therapies.

While αβ T cell-based platforms dominate current clinical development, their reliance on extensive genome engineering to mitigate alloreactivity highlights intrinsic limitations. In parallel, alternative immune cell subsets are being actively explored for allogeneic applications, leveraging their unique biological properties to bypass key barriers such as GvHD, immune rejection, and limited tumor infiltration. These include γδ T cells, iNKT cells, MAIT cells, and iPSC-derived immune effectors. Collectively, these platforms expand the design space of allogeneic therapies by offering distinct advantages and trade-offs that are increasingly informing the design of next-generation cell therapies [7] (Figure 2).

### 4.1. γδ T Cells: MHC-Independent Recognition and Innate-like Cytotoxicity

γδ T cells represent a compelling platform for allogeneic cell therapy due to their ability to recognize target cells in a MHC-independent manner. Unlike conventional αβ T cells, γδ T cells respond to stress-induced ligands, phosphoantigens, and metabolic dysregulation, enabling broad tumor recognition without reliance on classical antigen presentation [11,12,56,57]. This intrinsic biology reduces the risk of GvHD and minimizes the need for TCR gene editing, supporting their development as “off-the-shelf” therapies. Mechanistically, γδ T cells bridge innate and adaptive immunity through recognition of phosphoantigens via butyrophilin molecules (e.g., BTN3A1) and stress-associated ligands such as MICA, MICB, and UL16-binding proteins, as well as tumor-associated targets including Annexin A2 and EphA2 [58,59]. These features enable rapid cytotoxicity, cytokine production, and tissue surveillance, particularly within epithelial compartments, thereby addressing key limitations of conventional T cell therapies in solid tumors [11].

Early clinical translation has established proof-of-concept for the safety and feasibility of allogeneic γδ T cell therapy. A first-in-human Phase I study of haploidentical donor-derived Vγ9Vδ2 T cells in patients with relapsed or refractory acute myeloid leukemia demonstrated a favorable safety profile, with no dose-limiting toxicities, cytokine release syndrome, or GvHD observed, alongside preliminary evidence of anti-leukemic activity, including complete remission in one patient and transient disease control in others [60]. Although limited by small cohort size, these findings provided an important clinical foundation supporting the use of donor-derived γδ T cells without extensive genetic modification. Building on this foundation, the clinical landscape has rapidly evolved toward engineered and scalable platforms as shown in Table 2.

CAR-modified γδ T cell therapies are now being evaluated across hematologic malignancies and solid tumors, exemplified by anti-CD20 CAR-γδ T cells (ADI-001; NCT04735471), reflecting the broader emergence of programmable γδ platforms with enhanced persistence and resistance to immunosuppression. Within oncology, the therapeutic scope of allogeneic γδ T cells is extending from hematological malignancies to the immunosuppression of solid tumors [56,58]. Their intrinsic tissue-homing capacity, particularly of Vδ1 subsets, may support trafficking to epithelial and inflamed tumor sites, while their MHC-independent recognition of stress-associated ligands provides an opportunity to target tumors with impaired classical antigen presentation [66,67]. Current oncology-directed trials therefore leverage γδ T cells both as naturally cytotoxic effectors and as engineered CAR-bearing platforms [68,69].

Nevertheless, several challenges persist, including variability in ex vivo expansion, subset heterogeneity, and limited in vivo persistence. Ongoing engineering strategies, including cytokine armoring, metabolic reprogramming, and resistance to inhibitory signaling, aim to enhance durability and therapeutic efficacy, further positioning γδ T cells as a next-generation allogeneic cell therapy platform.

### 4.2. iNKT Cells: Invariant Recognition and Reduced Alloreactivity

iNKT cells constitute a distinct T cell subset characterized by a semi-invariant TCR that recognizes glycolipid antigens presented by the monomorphic molecule CD1d. This restricted recognition mechanism confers a reduced risk of alloreactivity, making iNKT cells an attractive candidate for allogeneic therapy [14,70]. Beyond their safety profile, iNKT cells exhibit potent immunomodulatory functions, including the ability to secrete large quantities of cytokines and recruit other immune effectors. Preclinical studies have shown that CAR-engineered iNKT cells can mediate effective antitumor activity while maintaining a low risk of GvHD [15]. Early clinical translation further supports this safety profile (Table 2). A Phase I study of CD19-targeted CAR-iNKT cells in patients with relapsed or refractory B cell malignancies (NCT00840853) demonstrated the feasibility of this approach, with no observed GvHD and encouraging preliminary signals of clinical activity [71]. More broadly, emerging clinical data suggest that CAR-iNKT platforms may combine targeted cytotoxicity with intrinsic immunoregulatory functions, enabling both direct tumor killing and modulation of the TME [72].

To address manufacturing limitations associated with the low frequency of circulating iNKT cells, iPSC-derived iNKT platforms are being actively developed. iPSC technology enables the generation of clonal, renewable master cell banks that can be differentiated into homogeneous iNKT cell populations, supporting scalable, off-the-shelf production. A first-in-human Phase I study of allogeneic iPSC-derived iNKT cells in patients with recurrent head and neck cancer demonstrated a favorable safety profile, with limited dose-limiting toxicity and early evidence of immune activation and disease stabilization, providing clinical proof-of-concept for scalable, off-the-shelf iNKT platforms [73].

Important challenges continue to limit clinical translation, including optimization of differentiation protocols, maintenance of functional maturity, and long-term in vivo persistence. Nevertheless, the convergence of CAR engineering and iPSC-based manufacturing is positioning iNKT cells as a highly versatile and scalable platform for allogeneic immunotherapy. In addition to their low risk of GvHD, iNKT cells possess intrinsic immunoregulatory properties that may contribute to the modulation of the TME, further enhancing their therapeutic potential in allogeneic settings.

### 4.3. MAIT Cells: Tissue Homing and Antimicrobial-like Recognition

MAIT cells are an innate-like T cell population that recognizes metabolite-derived antigens presented by the highly conserved MR1 molecule. Similar to iNKT cells, this monomorphic restriction reduces the likelihood of alloreactivity, supporting their application in allogeneic settings [14,74]. MAIT cells are enriched in mucosal tissues and exhibit strong tissue-homing capabilities, which may be advantageous for targeting solid tumors. In addition, they display rapid effector responses, including cytotoxicity and cytokine production, and may retain functional activity under certain immunosuppressive conditions within the TME, further enhancing their therapeutic appeal. Preclinical studies have established proof-of-concept for the use of MAIT cells as an allogeneic cell therapy platform. MAIT cells can be isolated from peripheral blood, expanded using MR1 ligands such as 5-OP-RU, and genetically engineered to express CARs. In a seminal study, CAR-MAIT cells targeting CD19 and HER2 showed antigen-specific cytotoxicity against tumor cell lines, with functional activity comparable to conventional CAR-T cells in vitro [75]. Additional studies have shown that MAIT cells can be engineered with CAR constructs targeting antigens such as mesothelin, further supporting the versatility of this platform [76].

However, the role of MAIT cells in cancer is not yet fully understood, with some studies suggesting context-dependent pro- or anti-tumor functions [77]. This functional plasticity represents both an opportunity and a challenge for therapeutic development, particularly in the context of allogeneic applications. Furthermore, current evidence for CAR-MAIT therapies remains largely limited to preclinical studies, with limited in vivo validation and no established clinical data to date. Key challenges include defining optimal expansion and engineering strategies, ensuring functional stability, and determining the tumor contexts in which MAIT cell-based therapies can provide the greatest clinical benefit. These properties position MAIT cells as a promising but still emerging platform for allogeneic immunotherapy, with unique advantages in tissue targeting and innate-like immune recognition that warrant further investigation [78].

### 4.4. iPSC-Derived Immune Cells: Scalable and Programmable Platforms

iPSC-derived immune cells represent a highly versatile and programmable platform for allogeneic therapy. Unlike primary cell sources, iPSCs enable virtually unlimited expansion and clonal selection, allowing precise genetic modification at the stem cell stage prior to differentiation into effector cells [4,79]. iPSC-derived platforms have been used successfully to generate CAR-T cells, NK cells, and iNKT cells with defined phenotypes and enhanced functional properties. This approach facilitates the integration of multiplex genome editing, including HLA modulation and insertion of synthetic circuits, within a controlled and standardized manufacturing process [80]. This platform therefore addresses a fundamental bottleneck in allogeneic therapy by improving product consistency and enabling scalable, off-the-shelf manufacturing. As such, iPSC-based systems represent a shift from donor-dependent to fully programmable cellular therapies.

However, several challenges must be addressed to fully realize their clinical potential, including ensuring complete and stable differentiation, avoiding residual pluripotent cells, and achieving in vivo persistence comparable to primary immune cells. In addition, regulatory considerations and manufacturing complexity also represent barriers to widespread clinical adoption. Nevertheless, these features position iPSC platforms as a central component of next-generation allogeneic cell therapy development.

## 5. Cross-Platform Comparison and Translational Positioning

The expanding diversity of allogeneic cell platforms requires a systematic framework for comparative evaluation and clinical positioning. While conventional αβ T cells offer well-established clinical efficacy, alternative platforms such as γδ T cells, iNKT cells, and MAIT cells provide intrinsic immune compatibility. These platforms also exhibit tissue-targeting properties and often require less extensive engineering. In contrast, iPSC-derived systems address scalability and standardization but introduce additional manufacturing and regulatory complexity. These trade-offs highlight that no single platform is universally optimal, and that platform selection must be guided by disease context and therapeutic objectives. This perspective aligns with the emerging paradigm of platform-informed engineering, in which the biological characteristics of each cell type are leveraged to minimize engineering burden while maximizing therapeutic efficacy [7]. Accordingly, the therapeutic potential is ultimately defined by a combination of persistence, safety, engineering feasibility, and suitability for specific disease contexts, rather than any single defining feature.

### 5.1. Persistence and Tumor Targeting

Persistence is a key determinant of clinical efficacy. Autologous αβ CAR-T cells have exhibited the capacity for long-term engraftment and durable remissions, establishing persistence as a key benchmark for therapeutic success [1,2]. In contrast, allogeneic αβ T cell therapies frequently exhibit limited persistence due to immune-mediated rejection, resulting in transient responses despite initial tumor clearance [7]. Alternative platforms display distinct persistence profiles. γδ T cells and innate-like subsets such as iNKT and MAIT cells typically exhibit shorter in vivo lifespans but may compensate through rapid effector responses and reduced susceptibility to exhaustion [11,14]. iPSC-derived products offer the potential for controlled differentiation into memory-like states, although their long-term persistence in clinical settings has yet to be fully established [4].

In solid tumors, persistence alone is insufficient to ensure efficacy. Therapeutic success is further constrained by impaired trafficking, limited tumor infiltration, and the suppressive TME [16]. Conventional αβ T cells are particularly vulnerable to these barriers. In contrast, innate-like subsets possess intrinsic tissue-homing properties and can respond to stress-associated signals, potentially enhancing tumor localization and activity. γδ T cells, for example, recognize epithelial stress ligands, while MAIT and iNKT cells are enriched in peripheral tissues, supporting their relevance in solid tumor contexts [11]. Engineering strategies, including chemokine receptor modulation and resistance to inhibitory signaling pathways, can further enhance tumor targeting, particularly in iPSC-derived platforms. Persistence and tumor accessibility represent coupled determinants of efficacy, with their relative importance varying across disease settings.

### 5.2. Safety, Alloreactivity, and Engineering Complexity

Alloreactivity is a defining challenge in allogeneic cell therapy and a key differentiator between platforms. Conventional αβ T cells carry an inherent risk of GvHD due to MHC-restricted TCR recognition, necessitating extensive genome editing to eliminate endogenous TCR expression [20,81]. Clinical data indicate that such modifications can effectively mitigate GvHD, although they add complexity to product design and manufacturing [25]. In contrast, γδ T cells, iNKT cells, and MAIT cells exhibit intrinsically low alloreactive potential due to their MHC-independent or monomorphic antigen recognition mechanisms. This inherent safety profile reduces the need for extensive genetic manipulation and potentially improves translational feasibility. iPSC-derived platforms offer the potential for more comprehensive immune evasion through multiplex engineering, including HLA modulation and checkpoint regulation. However, these approaches raise additional concerns related to genomic stability, long-term safety, and regulatory complexity [80].

Engineering burden is a key determinant of scalability and clinical translation. Allogeneic αβ T cell platforms typically require multiple genetic modifications, including TCR disruption, HLA modulation, and CAR insertion, often achieved through multiplex genome editing [54,82]. While increasingly feasible, these approaches add layers of complexity that can impact manufacturing efficiency, cost, and regulatory approval. Alternative platforms may reduce this engineering burden. γδ T cells and innate-like subsets often require fewer modifications due to their favorable safety profiles, although challenges related to expansion and consistency persist. In contrast, iPSC-derived systems shift complexity upstream, enabling extensive genetic engineering at the stem cell stage followed by standardized differentiation into effector cells [79,80]. Thus, manufacturability is not solely determined by the number of genetic modifications but also by the overall architecture of the production process, including scalability, reproducibility, and quality control.

Safety in allogeneic cell therapy reflects a balance between intrinsic immune compatibility and the extent of required engineering. This interplay ultimately shapes the feasibility of large-scale manufacturing and successful clinical translation across different platforms.

### 5.3. Integrated Platform Positioning

These comparative dimensions define a multidimensional landscape in which each platform occupies a distinct position (Figure 2). Conventional αβ T cells are the most clinically validated approach, particularly for hematological malignancies, but require extensive engineering to address alloreactivity and immune rejection. γδ T cells and innate-like subsets offer simplified safety profiles and may be better suited for applications requiring efficient tissue targeting although their clinical development is at an earlier stage. iPSC-derived systems provide unmatched scalability and programmability, albeit with increased manufacturing and regulatory complexity. This analysis supports a context-dependent model of platform selection, in which therapeutic design is guided by disease-specific requirements rather than a one-size-fits-all approach. Future advances will likely emerge from hybrid strategies that combine favorable biological properties with targeted engineering solutions, ultimately converging on optimized, indication-specific allogeneic cell therapy platforms.

## 6. Engineering Strategies Shaping Next-Generation Allogeneic T Cell Platforms

**Figure 3:**
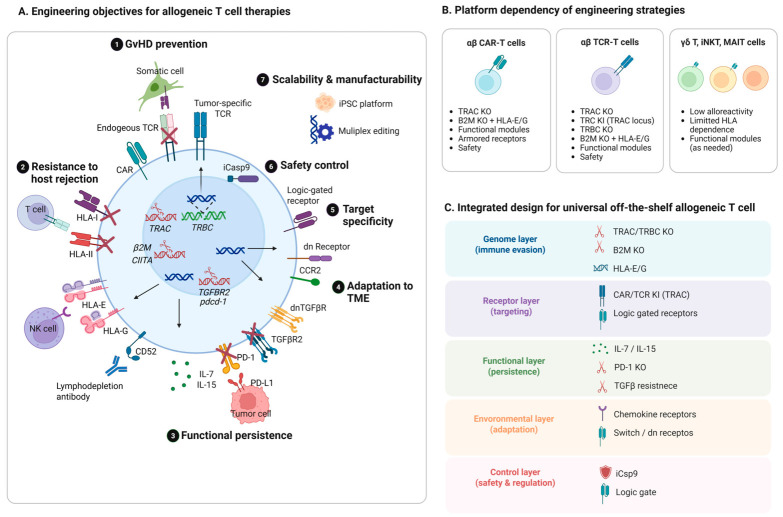
Engineering framework and platform-dependent strategies for allogeneic T cell therapies. Conceptual framework outlining key engineering objectives, platform-specific requirements, and integrated design principles for next-generation allogeneic T cell therapies. (**A**) Major engineering goals include prevention of graft-versus-host disease through endogenous TCR disruption, resistance to host immune rejection through HLA modulation and immune-evasive strategies, enhancement of functional persistence, adaptation to the TME, improved target specificity, safety control, and scalable manufacturing using approaches such as multiplex genome editing and iPSC-based platforms. (**B**) Platform-sepcific engineering requirements are compared across αβ CAR-T, αβ TCR-T, and innate-like T cell platforms, including TCR disruption or knock-in, B2M knock-out with HLA-E/G expression, functional modules, armored receptors, and safety-control strategies. (**C**) Integrated design model for universal off-the-shelf allogeneic T cell therapy, organized into genome, receptor, functional, environmental, and control layers that collectively regulate immune evasion, targeting, persistence, TME adaptation, and safety.

Therapeutic performance of allogeneic cell platforms is driven not by cellular origin alone, but by shared engineering solutions that address key biological constraints. Across diverse platforms, including αβ T cells, γδ T cells, iNKT cells, MAIT cells, and iPSC-derived effectors, a common set of design objectives has emerged. These include prevention of alloreactivity, evasion of host immune rejection, enhancement of persistence and functional fitness, adaptation to the TME, and incorporation of controllable safety mechanisms. Rather than representing isolated modification, these strategies define an integrated and modular engineering framework in which individual design elements are combined to address specific biological constraints, enabling rational development of off-the-shelf cellular therapies (Figure 3).

A critical design principle is that each engineering intervention introduces compensatory risks. For example, strategies designed to reduce host immune recognition can simultaneously create new vulnerabilities that require compensatory engineering solutions. Suppression of HLA-mediated recognition may diminish host T cell responses but can also increase susceptibility to innate immune surveillance [7]. IL-15 or IL-7 armoring can enhance expansion and memory-like persistence but may also increase inflammatory toxicity or uncontrolled proliferation if expression is not tightly regulated [83]. PD-1 disruption may improve resistance to exhaustion, yet constitutive checkpoint removal can reduce physiological control of activated cells [84]. Finally, multiplex genome editing enables immune evasion and receptor optimization but increases concerns regarding off-target editing, chromosomal rearrangements, clonal selection, release testing, and long-term genomic stability [85]. Therefore, engineering strategies should be evaluated as coupled design modules rather than independent improvements.

### 6.1. Engineering Immune Compatibility and Evasion

A central requirement for allogeneic therapies is the separation of antigen-specific cytotoxicity from endogenous alloreactive signaling. In conventional αβ T cell platforms, this is most commonly achieved through targeted disruption of the TCR, typically via knock-out of the TRAC or TCR β constant (TRBC) loci using genome editing technologies such as CRISPR-Cas9 or TALENs [54,81]. This strategy effectively eliminates MHC-restricted recognition of host tissues and mitigates the risk of GvHD, functionally converting T cells into synthetic effector cells whose specificity is defined by the introduced receptor. In addition to TRAC disruption, knock-out of the TRBC locus has been employed to prevent mispairing between endogenous and introduced TCR chains, thereby improving receptor fidelity and reducing the risk of off-target reactivity. Furthermore, targeted knock-in of transgenic TCRs into the TRAC locus has emerged as a refined engineering approach, enabling physiological regulation of TCR expression under the endogenous promoter while simultaneously eliminating native TCR expression. This strategy has been shown to enhance functional potency, reduce tonic signaling, and improve the safety profile of engineered TCR-T cells in both preclinical and clinical settings [54,86].

Beyond prevention of GvHD, successful allogeneic therapies must also evade HvG responses to achieve durable persistence. Disruption of B2M to abrogate HLA class I expression is widely employed to reduce recognition by host CD8^+^ T cells [82]. However, a complete loss of HLA class I renders engineered cells susceptible to NK cell-mediated cytotoxicity via “missing-self” recognition. To mitigate this vulnerability, engineered cells are frequently modified to express non-classical HLA molecules, such as HLA-E or HLA-G, which engage inhibitory NK cell receptors (e.g., NKG2A) and suppress cytotoxic activation [80,87]. In TCR-engineered platforms, disruption of class II transactivator (CIITA) can further reduce CD4^+^ T cell–mediated recognition by limiting HLA class II expression [18,86]. Additional strategies, including overexpression of CD47, further reduce phagocytic clearance by macrophages [88]. Complementing these cell-intrinsic approaches, pharmacologic control of host immunity can be achieved through disruption of the CD52 antigen. CD52 is the target of alemtuzumab, which is used in lymphodepleting regimens to eliminate host lymphocytes. Knock-out of CD52 renders engineered cells resistant to alemtuzumab-mediated depletion, enabling selective suppression of host immune cells while preserving the infused product. This strategy creates a transient window that limits HvG-mediated rejection and supports in vivo expansion. CD52 disruption is frequently combined with TCR knock-out in allogeneic platforms, as exemplified by UCART19 and related products, where alemtuzumab-containing conditioning has been associated with improved engraftment and persistence [26,81]. The extent of required immune evasion varies across platforms. γδ T cells, iNKT cells, and MAIT cells exhibit intrinsically reduced alloreactivity due to MHC-independent or monomorphic antigen recognition, thereby lowering the need for extensive TCR modification [11,14]. Conversely, iPSC-derived platforms enable extensive pre-programmed immune cloaking through multiplex genome editing at the clonal stage. Thus, immune evasion strategies must be tailored to the biological context of each platform rather than applied uniformly.

### 6.2. Enhancing Persistence and Functional Fitness

As highlighted in Section 5, persistence and tumor targeting are key determinants of therapeutic efficacy. Engineering strategies have therefore focused on enhancing both intrinsic cellular fitness and resistance to extrinsic suppressive signals. One widely adopted approach is targeted integration of synthetic receptors into defined genomic loci, such as the TRAC locus, which promotes uniform receptor expression and reduces tonic signaling, thereby limiting exhaustion [54]. This strategy has been exemplified in both CAR-T and TCR-T settings, where site-specific knock-in improves functional consistency compared to randomly integrated constructs. Cytokine support modules, including interleukin-7 (IL-7) and IL-15, enhance survival, proliferation and the acquisition of memory-like phenotypes [89,90]. For example, IL-15–expressing CAR constructs, including GD2-targeting CAR-iNKT cells, have shown enhanced in vivo persistence and antitumor activity, highlighting the role of cytokine autocrine support in sustaining functional fitness [91]. In parallel, engineering strategies that favor less differentiated states, including stem cell memory (T_SCM_) phenotypes, are associated with improved long-term persistence and proliferative capacity [92]. To sustain effector function under chronic antigen exposure, genetic disruption or modulation of inhibitory pathways has also been explored. PD-1 disruption can reduce exhaustion-associated signaling and improve T cell persistence, but permanent loss of PD-1 signaling may also impair control of excessive T cell activation and raise safety concerns [93]. Targeting immunosuppressive cytokine signaling pathways represents a complementary approach. For instance, disruption of TGF-β signaling through knock-out of TGF-β receptor 2 (TGFBR2) in CAR-T cells, or expression of dominant-negative TGF-β receptors in TCR-engineered T cells, has been shown to confer resistance to TME-mediated suppression and enhance effector function [94,95].

Beyond intrinsic fitness, effective tumor targeting requires adaptation to the TME. Engineering strategies such as expression of chemokine receptors matched to tumor-derived chemokines (for example, CCR2 or CXCR2), as well as dominant-negative or switch receptors that convert inhibitory signals into activating cues, can improve trafficking, infiltration, and functional resilience [17,96,97]. These approaches are being extended across alternative immune cell platforms, including γδ T cell-based CAR therapies, which combine innate-like recognition with engineered targeting to enhance persistence and function in solid tumor settings. Importantly, baseline persistence differs across platforms. Innate-like T cells may rely on rapid effector function rather than long-term engraftment, whereas stem cell-derived products offer opportunities for controlled differentiation and sustained activity. These differences reinforce the need to tailor persistence-enhancing strategies to platform-specific biology and disease context.

### 6.3. Safety Control and Regulatory Design

Given the potential for severe toxicities, including cytokine release syndrome, off-tumor effects and unintended alloreactivity, the incorporation of controllable safety mechanisms is an essential engineering requirement. Inducible suicide switches, such as inducible caspase 9 (iCasp9), provide a validated approach for rapid pharmacological elimination of infused cells in the event of adverse reactions [98]. More advanced strategies include logic-gated receptor systems that require combinatorial antigen recognition to trigger activation, thereby improving tumor specificity and reducing off-target toxicity [99]. In addition, transient expression platforms, such as mRNA-based engineering, provide an additional layer of control by limiting the duration of cellular activity, which may be particularly relevant in settings where long-term persistence is not needed.

The extent of safety engineering required varies across platforms. While innate-like T cells exhibit lower alloreactive potential, their cytokine profiles and in vivo behavior still necessitate robust control mechanisms. Accordingly, safety design must be integrated with functional engineering to ensure an appropriate balance between efficacy and risk.

### 6.4. Multiplex Engineering and Integrated Design Frameworks

The increasing complexity of allogeneic T cell therapy has driven the adoption of multiplex genome editing strategies, enabling the simultaneous modification of multiple genetic loci within a single cellular product. These approaches typically combine disruption of endogenous TCR expression, modulation of HLA molecules, targeted insertion of synthetic receptors, and incorporation of cytokine or checkpoint regulatory elements [7,45,54,81]. Such combinatorial engineering enables coordinated control of immune compatibility, resistance to host rejection and functional activity, forming the foundation of next-generation allogeneic platforms. These advances have catalyzed the emergence of “universal” CAR-T cells, in which multiplex genome engineering is used to minimize alloreactivity while enabling standardized, off-the-shelf deployment across patient populations. In this context, “universal” encompasses not only donor independence but also the integration of immune-evasive strategies, such as B2M disruption coupled with expression of non-classical HLA molecules to mitigate NK cell-mediated clearance, alongside functional enhancements including cytokine support and checkpoint modulation. Early clinical and translational studies support the feasibility of this approach, although durable engraftment and long-term safety continue to pose key challenges [41]. Beyond static genetic modifications, advances in synthetic biology are enabling the development of programmable cellular circuits that dynamically respond to environmental cues. These include inducible cytokine expression systems, logic-gated chimeric antigen receptors and signal-processing networks capable of integrating multiple inputs to control cellular behavior. Recent studies have revealed the use of synthetic genetic switches to achieve spatiotemporal control of CAR-T cell activity, as well as increasingly sophisticated circuit designs that enhance specificity and safety in complex tumor environments [100,101]. iPSC-derived platforms are particularly well suited to such strategies, as extensive genetic engineering can be performed at the clonal stage prior to differentiation, enabling precise and reproducible control over final product characteristics. However, similar design principles are increasingly being applied to primary T cell platforms, reflecting a broader shift towards programmable and adaptable therapeutic systems.

These engineering strategies define core design axes that enable systematic comparison and optimization of allogeneic platforms: (1) immune compatibility, (2) resistance to host rejection, (3) persistence and functional durability, (4) tumor targeting and microenvironmental adaptation, (5) safety control, and (6) manufacturability. This framework supports a transition from platform-driven to design-driven development, in which intrinsic cellular properties are integrated with modular engineering strategies. Future progress will likely depend on the rational coordination of these parameters to generate optimized, indication-specific and scalable off-the-shelf cell therapies, bringing the field closer to the realization of broadly applicable universal T cell platforms.

## 7. Translational and Manufacturing Challenges

Despite rapid advances in genome engineering platform design, the clinical translation of allogeneic T cell therapies is constrained by practical limitations that extend beyond biological optimization. Unlike autologous approaches, which are inherently patient-specific, allogeneic platforms must achieve consistency, scalability and broad applicability while navigating complex safety considerations. These constraints introduce interconnected challenges spanning manufacturing, regulatory evaluation, clinical implementation, and cost-effectiveness, ultimately influencing product design, clinical performance and commercial viability.

### 7.1. Manufacturing Scalability and Regulatory Constraints

A central promise of allogeneic cell therapy is the ability to generate large, standardized batches of cellular products from healthy donors or renewable sources. However, achieving true scalability while preserving cellular quality is technically demanding. Manufacturing workflows must accommodate donor variability, maintain cell fitness during ex vivo expansion, and ensure consistency across batches, all of which are critical for reproducible clinical outcomes [7,8]. The incorporation of genome editing further complicates manufacturing processes. Multiplex modifications, such as TCR disruption, HLA engineering and transgene insertion, can reduce yield, impair viability and increase product heterogeneity [54,81]. In addition, extensive manipulation may induce cellular stress, senescence or altered differentiation states, thereby compromising in vivo performance [102]. Cryopreservation, a key requirement for off-the-shelf deployment, further impacts cell quality, as freeze–thaw cycles can reduce viability, proliferative capacity and trafficking potential [90].

These technical challenges are closely coupled with regulatory considerations. Allogeneic T cell products incorporating genome editing technologies face heightened regulatory scrutiny regarding off-target effects, chromosomal rearrangements, insertional mutagenesis and long-term genomic stability [45,103]. The use of multiplex editing further amplifies these risks, necessitating rigorous characterization at both genomic and functional levels. Regulatory expectations increasingly include comprehensive assessment of the following: (1) off-target effects using unbiased genome-wide methods, (2) clonal diversity and expansion of aberrant subpopulations, (3) persistence and biodistribution of infused cells, and (4) risks associated with transgene integration. Additional complexity arises from advanced engineering features, such as logic-gated receptors or inducible systems, which require demonstration of predictable and controlled behavior in vivo [99]. iPSC-derived products introduce further regulatory challenges, including the risk of residual pluripotent cells and tumorigenicity, despite their advantages in clonal engineering and scalability. These factors highlight a fundamental trade-off between engineering sophistication and manufacturability, emphasizing the need for streamlined processes that balance innovation with regulatory feasibility [88]. Moreover, harmonization of regulatory standards across major jurisdictions may facilitate the global development, comparability, and evaluation of advanced gene-engineered immune cell therapies [104].

### 7.2. Clinical and Economic Limitations

Beyond manufacturing and regulatory considerations, the clinical evaluation of allogeneic T cell therapies presents distinct challenges. In contrast to autologous approaches, which are tailored to individual patients, allogeneic therapies must demonstrate consistent efficacy across diverse genetic and immunological backgrounds. Variability in HvG responses, immune-mediated rejection, and persistence contributes to heterogeneous clinical outcomes and complicates the interpretation of therapeutic efficacy [7,45]. Clinical trial design is further challenged by multiple confounding variables, including heterogeneous patient populations, differences in prior treatment, and variability in lymphodepleting conditioning regimens that influence both engraftment and toxicity [7]. Limited persistence of allogeneic cells complicates the assessment of response durability, while the absence of standardized endpoints in early-phase studies hinders cross-study comparisons. In addition, distinguishing between treatment failure due to insufficient intrinsic potency and failure due to immune-mediated rejection is challenging, underscoring the need for robust biomarkers that can reliably track engraftment, persistence, and immune clearance in vivo [8].

Economic considerations add an additional layer of complexity. Although allogeneic therapies are conceptually positioned as cost-effective alternatives to autologous approaches, this advantage has not yet been fully realized. The integration of genome editing, stringent quality control requirements and specialized manufacturing infrastructure contribute to high production costs [7,105]. Furthermore, the need to meet release criteria applicable to a broad patient population increases the burden of testing and validation. Clinical factors, including the requirement for lymphodepleting conditioning regimens and the potential need for repeat dosing due to limited persistence, may offset logistical advantages [7]. As a result, the cost-effectiveness and clinical positioning of allogeneic therapies are highly context-dependent and not yet well defined. Future clinical development will likely require adaptive trial designs, biomarker-driven patient stratification, and combination strategies with immune-modulating agents to enhance efficacy and durability. More broadly, addressing these challenges will require coordinated advances in manufacturing technologies, regulatory frameworks, and clinical methodologies. These considerations underscore a central tension in the field: the need to balance engineering complexity with scalability, safety, and real-world applicability to fully realize the potential of off-the-shelf T cell therapies.

## 8. Conclusions and Future Perspectives

Allogeneic T cell therapies have rapidly transitioned from theoretical concepts to clinically emerging platforms, supported by advances in genome engineering, synthetic biology, and cell manufacturing. Across both conventional αβ T cell-based systems and emerging innate-like platforms, these approaches expand the therapeutic landscape of off-the-shelf immunotherapy.

Despite this progress, broader clinical implementation remains shaped by fundamental challenges, including immune incompatibility, limited persistence, TME constraints, and manufacturing scalability. Importantly, the convergence of diverse platforms highlights that therapeutic performance is determined not solely by cellular origin, but by how effectively key design parameters, such as immune evasion, functional fitness, tumor targeting, and safety, are coordinated.

Looking forward, the field is shifting from incremental, platform-specific optimization toward integrated, design-driven engineering frameworks. Future strategies will increasingly rely on multiplex genome editing and synthetic biology to enable precise, programmable cellular functions, improving control over transgene expression while minimizing unintended interactions such as TCR mispairing. In parallel, aligning engineering approaches with platform-specific biology, particularly in innate-like lymphocyte populations, may reduce engineering burden and enhance scalability.

Advances in manufacturing, including standardized and stem cell–derived platforms, will further support reproducibility and broader clinical accessibility. Ultimately, the future of allogeneic T cell therapy lies in the rational integration of biology and engineering to deliver truly universal, programmable, and clinically scalable cell therapies.

## Figures and Tables

**Table 1:** Clinical studies of allogeneic CAR-T and TCR-T platforms in oncology.

Cell Type	Disease Category	Target	Product Name	Indication	Genome Editing Method	Phase Stage	Current Status	Trial No.	Trial Name	Ref.
CAR-T	Hematologic malignancy	BCMA	BCMA-UCART	MM	TALEN	Phase 1	Terminated	NCT03752541		-
			ALLO-715	R/R MM	TALEN	Phase 1	Not recruiting	NCT04093596	UNIVERSAL	[23]
			CYAD-211	R/R MM	miRNA based-shRNA	Phase1	Not recruiting	NCT04613557	IMMUNICY-1	[24]
		CD19	UCART19	R/R B-ALL (adult)	TALEN	Phase1	Completed	NCT02746952	CALM	[25]
			UCART19	R/R B-ALL (pediatric)	TALEN	Phase1	Terminated	NCT02808442	PALL	[26]
			ALLO-501	R/R LBCL or FL	TALEN	Phase1	Completed	NCT03939026	ALPHA	[27]
			ALLO-501A	R/R LBCL, CLL or SLL	TALEN	Phase1/2	Not recruiting	NCT04416984	ALPHA2	[28]
			CTX110	R/R B-ALL or NHL	CRISPR/Cas9	Phase1/2	Terminated	NCT04035434	CARBON	[29]
			alloCART-19	B-ALL	CRISPR/Cas9	Phase1	Recruiting	NCT04173988		-
			CB-010	B-NHL	ChrDNA/Cas9	Phase1	Recruiting	NCT04637763	ANTLER	[30]
		CD19/CD22	CTA101	R/R B-ALL or NHL	CRISPR/Cas9	Phase1	Recruiting	NCT04227015		[31]
		CD7	BE-CAR7	R/R T-CLL (pediatric)	Base-editing	Phase1	Recruiting	NCT05397184	TvT CAR7	[32]
		CD20/CD22	UCART20x22	R/R B-NHL	TALEN	Phase1/2	Recruiting	NCT05607420	NatHaLi-01	[33]
		CD22	UCART22	R/R B-ALL	TALEN	Phase1/2	Recruiting	NCT04150497	BALLI-01	[34]
		CD33	CART-33	R/R CD33^+^ AML	TALEN	Phase1	Terminated	NCT02799680		-
		CD70	CTX130	R/R TCM or BCM	CRISPR/Cas9	Phase1	Terminated	NCT04502446	COBALT-LYM	[35]
		CD123	UCART123v1.2	R/R AML	TALEN	Phase1	Recruiting	NCT03190278	AMELI-01	[36]
	Solid tumor	CD70	CTX130	a/R/R ccRCC	CRISPR/Cas9	Phase1	Terminated	NCT04438083	COBALT-RCC	[37]
			ALLO-316	a/m ccRCC	CRISPR/Cas9	Phase1	Recruiting	NCT04696731	TRAVERSE	[38]
		MUC1	P-MUC1C-ALLO1	Solid tumor	Cas/CLOVER	Phase1/2	Not recruiting	NCT05239143		[39]
TCR-T	Hematologic malignancy	HA-1, HA-2	TSC-100, TSC-101	AML, MDS, ALL	Lentiviral vector	Phase1	Recruiting	NCT05473910	ALLOHA	[40]

(1) ALL—acute lymphoblastic leukemia; (2) a/m—advanced or metastatic; (3) AML—acute myeloid leukemia; (4) a/R/R—advanced or relapsed/refractory; (5) BCM—B cell malignancy; (6) ccRCC—clear cell renal cell carcinoma; (7) CLL—chronic lymphocytic leukemia; (8) FL—follicular lymphoma; (9) LBCL—large B cell lymphoma; (10) MM—multiple myeloma; (11) NHL—non-Hodgkin lymphoma; (12) R/R—relapsed or refractory; (13) SLL—small lymphocytic lymphoma; (14) TCM—T cell malignancy.

**Table 2:** Clinical studies of allogeneic γδ T and iNKT platforms in oncology.

Cell Type	Disease Category	Target	Product Name	Indication	Phase Stage	Current Status	Trial No.	Trial Name	Ref.
CAR-γδ T	Hematologic malignancy	B7-H3	UTAA06	R/R AML	Phase1	Unknown	NCT05731219		-
		BCMA	UTAA17	R/R MM	Not applicable	Recruiting	NCT06279026		[61]
		CD19		BCL, ALL, CLL	Phase1	Unknown	NCT02656147		-
			UTAA09	R/R HLM	Early phase1	Recruiting	NCT06092047		-
			HQ103	B-ALL	Phase1	Recruiting	NCT06056752		-
			UTAA09	R/R B cell NHL	Phase1	Recruiting	NCT06503211		-
		CD20	ADI-001	R/R BCM	Phase1	Recruiting	NCT04735471	GLEAN-1	[62]
		NKG2DL	CTM-N2D	Leukemia	Phase1	Recruiting	NCT05302037	ANGELICA	[63]
		Phosphoantigen	TCB008	AML, MDS	Phase2	Terminated	NCT05358808	ACHIEVE	-
	Solid tumor	B7-H3	HQ104	Brain glioma	Phase1/2	Recruiting	NCT06018363		-
			UTAA06	B7-H3^+^ R/R solid tumor	Phase1	Completed	NCT06372236		[64]
		CD70	ADI-270	R/R ccRCC	Phase1/2	Not recruiting	NCT06480565		[65]
		GD2	-	R/R or PD NB, R/R OS	Phase1	Recruiting	NCT05400603		-
		HLA-G	CAR001	R/R solid tumor	Phase1/2	Recruiting	NCT06150885		-
		NKG2DL	CTM-N2D	R/R solid tumor	Phase1	Unknown	NCT04107142		-
CAR-iNKT	Hematologic malignancy	CD19	-	ALL, CLL, NHL	Phase1	Not recruiting	NCT00840853	MULTIPRAT	[60]
			KUR-502	R/R B-ALL or CLL	Phase1	Recruiting	NCT03774654	ANCHOR	-
			KUR-502	R-NHL, BCL, B-ALL	Phase1	Recruiting	NCT05487651	ANCHOR2	-

(1) ALL—acute lymphoblastic leukemia; (2) a/m—advanced or metastatic; (3) AML—acute myeloid leukemia; (4) a/R/R—advanced or relapsed/refractory; (5) BCL—B cell lymphoma; (6) BCM—B cell malignancy; (7) ccRCC—clear cell renal cell carcinoma; (8) CLL—chronic lymphocytic leukemia; (9) HLM—hematolymphatic malignancy; (10) MDS—myelodysplastic syndrome; (11) NB—neuroblastoma; (12) NHL—non-Hodgkin lymphoma; (13) OS—osteosarcoma; (14) PD—progressive disease; (15) R/R—relapsed or refractory.

## Data Availability

No new data were created or analyzed in this study.

## References

[1] Maude S.L., Laetsch T.W., Buechner J., Rives S., Boyer M., Bittencourt H., Bader P., Verneris M.R., Stefanski H.E., Myers G.D. (2018). Tisagenlecleucel in Children and Young Adults with B-Cell Lymphoblastic Leukemia. N. Engl. J. Med..

[2] Neelapu S.S., Locke F.L., Bartlett N.L., Lekakis L.J., Miklos D.B., Jacobson C.A., Braunschweig I., Oluwole O.O., Siddiqi T., Lin Y. (2017). Axicabtagene Ciloleucel CAR T-Cell Therapy in Refractory Large B-Cell Lymphoma. N. Engl. J. Med..

[3] Lonez C., Breman E. (2024). Allogeneic CAR-T Therapy Technologies: Has the Promise Been Met?. Cells.

[4] Kawamoto H., Masuda K. (2024). Trends in cell medicine: From autologous cells to allogeneic universal-use cells for adoptive T-cell therapies. Int. Immunol..

[5] Lee H.-M. (2022). Strategies for Manipulating T Cells in Cancer Immunotherapy. Biomol. Ther..

[6] Lee S.W., Lee H.-M. (2024). Engineered T Cell Receptor for Cancer Immunotherapy. Biomol. Ther..

[7] Depil S., Duchateau P., Grupp S.A., Mufti G., Poirot L. (2020). ‘Off-the-shelf’ allogeneic CAR T cells: Development and challenges. Nat. Rev. Drug Discov..

[8] Levine B.L., Miskin J., Wonnacott K., Keir C. (2017). Global Manufacturing of CAR T Cell Therapy. Mol. Ther. Methods Clin. Dev..

[9] Abodunrin F., Olson D.J., Emehinola O., Bestvina C.M. (2025). Adopting tomorrow’s therapies today: A perspective review of adoptive cell therapy in lung cancer. Ther. Adv. Med. Oncol..

[10] Krakow E.F., Brault M., Summers C., Cunningham T.M., Biernacki M.A., Black R.G., Woodward K.B., Vartanian N., Kanaan S.B., Yeh A.C. (2024). HA-1-targeted T-cell receptor T-cell therapy for recurrent leukemia after hematopoietic stem cell transplantation. Blood.

[11] Silva-Santos B., Mensurado S., Coffelt S.B. (2019). γδ T cells: Pleiotropic immune effectors with therapeutic potential in cancer. Nat. Rev. Cancer.

[12] Schonefeldt S., Wais T., Herling M., Mustjoki S., Bekiaris V., Moriggl R., Neubauer H.A. (2021). The Diverse Roles of gammadelta T Cells in Cancer: From Rapid Immunity to Aggressive Lymphoma. Cancers.

[13] Shokati A., Sanjari-Pour M., Akhavan Rahnama M., Hoseinzadeh S., Vaezi M., Ahmadvand M. (2025). Allogeneic CART progress: Platforms, current progress and limitations. Front. Immunol..

[14] Godfrey D.I., Koay H.F., McCluskey J., Gherardin N.A. (2019). The biology and functional importance of MAIT cells. Nat. Immunol..

[15] Heczey A., Liu D., Tian G., Courtney A.N., Wei J., Marinova E., Gao X., Guo L., Yvon E., Hicks J. (2014). Invariant NKT cells with chimeric antigen receptor provide a novel platform for safe and effective cancer immunotherapy. Blood.

[16] Chen T., Wang M., Chen Y., Liu Y. (2024). Current challenges and therapeutic advances of CAR-T cell therapy for solid tumors. Cancer Cell Int..

[17] Rafiq S., Hackett C.S., Brentjens R.J. (2020). Engineering strategies to overcome the current roadblocks in CAR T cell therapy. Nat. Rev. Clin. Oncol..

[18] Ren J., Liu X., Fang C., Jiang S., June C.H., Zhao Y. (2017). Multiplex Genome Editing to Generate Universal CAR T Cells Resistant to PD1 Inhibition. Clin. Cancer Res..

[19] Lyu Z., Niu S., Fang Y., Chen Y., Li Y.R., Yang L. (2025). Addressing graft-versus-host disease in allogeneic cell-based immunotherapy for cancer. Exp. Hematol. Oncol..

[20] Ferrara J.L.M., Levine J.E., Reddy P., Holler E. (2009). Graft-versus-host disease. Lancet.

[21] Ljunggren H.G., Kärre K. (1990). In search of the ‘missing self’: MHC molecules and NK cell recognition. Immunol. Today.

[22] Tokarew N., Ogonek J., Endres S., von Bergwelt-Baildon M., Kobold S. (2019). Teaching an old dog new tricks: Next-generation CAR T cells. Br. J. Cancer.

[23] Mailankody S., Matous J.V., Chhabra S., Liedtke M., Sidana S., Oluwole O.O., Malik S., Nath R., Anwer F., Cruz J.C. (2023). Allogeneic BCMA-targeting CAR T cells in relapsed/refractory multiple myeloma: Phase 1 UNIVERSAL trial interim results. Nat. Med..

[24] Lonez C., Bolsee J., Huberty F., Nguyen T., Jacques-Hespel C., Anguille S., Flament A., Breman E. (2024). Clinical Proof-of-Concept of a Non-Gene Editing Technology Using miRNA-Based shRNA to Engineer Allogeneic CAR T-Cells. Int. J. Mol. Sci..

[25] Benjamin R., Jain N., Maus M.V., Boissel N., Graham C., Jozwik A., Yallop D., Konopleva M., Frigault M.J., Teshima T. (2022). UCART19, a first-in-class allogeneic anti-CD19 chimeric antigen receptor T-cell therapy for adults with relapsed or refractory B-cell acute lymphoblastic leukaemia (CALM): A phase 1, dose-escalation trial. Lancet Haematol..

[26] Benjamin R., Graham C., Yallop D., Jozwik A., Mirci-Danicar O.C., Lucchini G., Pinner D., Jain N., Kantarjian H., Boissel N. (2020). Genome-edited, donor-derived allogeneic anti-CD19 chimeric antigen receptor T cells in paediatric and adult B-cell acute lymphoblastic leukaemia: Results of two phase 1 studies. Lancet.

[27] Neelapu S.S., Nath R., Munoz J., Tees M., Miklos D.B., Frank M.J., Malik S.A., Stevens D., Shin C.R., Balakumaran A. (2021). ALPHA Study: ALLO-501 Produced Deep and Durable Responses in Patients with Relapsed/Refractory Non-Hodgkin’s Lymphoma Comparable to Autologous CAR T. Blood.

[28] Jallouk A.P., Ge Z., Kaimal V., Furmanak T., Sommer C., Lauron E.J., Sasu B.J., Neelapu S.S., Bachireddy P. (2023). Cellular Mechanisms Affecting Allogeneic CAR T Cell Expansion and Rejection in Large B-Cell Lymphoma. Blood.

[29] McGuirk J.P., Tam C.S., Kröger N., Riedell P.A., Murthy H.S., Ho P.J., Maakaron J.E., Waller E.K., Awan F.T., Shaughnessy P.J. (2022). CTX110 allogeneic CRISPR-Cas9-engineered CAR T cells in patients (Pts) with relapsed or refractory (R/R) large B-cell lymphoma (LBCL): Results from the phase 1 dose escalation carbon study. Blood.

[30] O’Brien S., Nastoupil L.J., Essell J., Dsouza L., Hart D., Matsuda E., Satterfield T., Nesheiwat T., Hammad A., Davi F. (2022). A first-in-Human phase 1, multicenter, open-label study of CB-010, a next-generation CRISPR-edited allogeneic anti-CD19 CAR-T cell therapy with a PD-1 knock-out, in patients with Relapsed/Refractory b cell non-Hodgkin lymphoma (ANTLER study). Blood.

[31] Hu Y., Zhou Y., Zhang M., Ge W., Li Y., Yang L., Wei G., Han L., Wang H., Yu S. (2021). CRISPR/Cas9-Engineered Universal CD19/CD22 Dual-Targeted CAR-T Cell Therapy for Relapsed/Refractory B-cell Acute Lymphoblastic Leukemia. Clin. Cancer Res..

[32] Chiesa R., Georgiadis C., Syed F., Zhan H., Etuk A., Gkazi S.A., Preece R., Ottaviano G., Braybrook T., Chu J. (2023). Base-Edited CAR7 T Cells for Relapsed T-Cell Acute Lymphoblastic Leukemia. N. Engl. J. Med..

[33] Abramson J.S., Ramakrishnan A., Pierola A.A., Braunschweig I., Cartron G., Thieblemont C., Pérez-Simón J.A., Barba P., Riedell P.A., Solano C. (2023). Preliminary results of nathali-01: A first-in-human phase I/IIa study of UCART20x22, a dual allogeneic CAR-T cell product targeting CD20 and CD22, in relapsed or refractory (R/R) non-hodgkin lymphoma (NHL). Blood.

[34] Jain N., Roboz G.J., Konopleva M., Liu H., Jabbour E., Poirot C., Schiffer-Manniou C., Gouble A., Haider A., Zernovak O. (2020). Preliminary results of Balli-01: A phase I study of UCART22 (allogeneic engineered T-cells expressing anti-CD22 chimeric antigen receptor) in adult patients with relapsed or refractory (R/R) CD22+ B-cell acute lymphoblastic leukemia (B-ALL). Blood.

[35] Iyer S.P., Sica R.A., Ho P.J., Prica A., Zain J., Foss F.M., Hu B., Beitinjaneh A., Weng W.-K., Kim Y.H. (2025). Safety and activity of CTX130, a CD70-targeted allogeneic CRISPR-Cas9-engineered CAR T-cell therapy, in patients with relapsed or refractory T-cell malignancies (COBALT-LYM): A single-arm, open-label, phase 1, dose-escalation study. Lancet Oncol..

[36] Roboz G.J., DeAngelo D.J., Sallman D.A., Guzman M.L., Desai P., Kantarjian H.M., Konopleva M., Bejanyan N., Elmariah H., Esteva F.J. (2020). Ameli-01: Phase I, open label dose-escalation and dose-expansion study to evaluate the safety, expansion, persistence and clinical activity of UCART123 (allogeneic engineered T-cells expressing anti-CD123 chimeric antigen receptor), administered in patients with relapsed/refractory acute myeloid leukemia. Blood.

[37] Pal S.K., Tran B., Haanen J., Hurwitz M.E., Sacher A., Tannir N.M., Budde L.E., Harrison S.J., Klobuch S., Patel S.S. (2024). CD70-Targeted Allogeneic CAR T-Cell Therapy for Advanced Clear Cell Renal Cell Carcinoma. Cancer Discov..

[38] Srour S., Chahoud J., Drakaki A., Curti B., Gibney G., Pal S., Tang L., Charmsaz S., Atwell J., Robbins P. (2025). ALLO-316 in advanced clear cell renal cell carcinoma (ccRCC): Updated results from the phase 1 TRAVERSE study. J. Clin. Oncol..

[39] Henry J., Oh D., Eskew J., Baranda J., Rivera I.I.R., Dumbrava E., Cohen E., Belani R., McCaigue J., Shedlock D. (2022). 728 Phase 1 study of P-MUC1C-ALLO1 allogeneic CAR-T cells in patients with epithelial-derived cancers. J. Immunother. Cancer.

[40] Qasim W., Zhan H., Samarasinghe S., Adams S., Amrolia P., Stafford S., Butler K., Rivat C., Wright G., Somana K. (2017). Molecular remission of infant B-ALL after infusion of universal TALEN gene-edited CAR T cells. Sci. Transl. Med..

[41] Locatelli F., Del Bufalo F., Quintarelli C. (2024). Allogeneic chimeric antigen receptor T cells for children with relapsed/refractory B-cell precursor acute lymphoblastic leukemia. Haematologica.

[42] Robbins P.F., Morgan R.A., Feldman S.A., Yang J.C., Sherry R.M., Dudley M.E., Wunderlich J.R., Nahvi A.V., Helman L.J., Mackall C.L. (2011). Tumor regression in patients with metastatic synovial cell sarcoma and melanoma using genetically engineered lymphocytes reactive with NY-ESO-1. J. Clin. Oncol..

[43] Rapoport A.P., Stadtmauer E.A., Binder-Scholl G.K., Goloubeva O., Vogl D.T., Lacey S.F., Badros A.Z., Garfall A., Weiss B., Finklestein J. (2015). NY-ESO-1-specific TCR-engineered T cells mediate sustained antigen-specific antitumor effects in myeloma. Nat. Med..

[44] Stadtmauer E.A., Fraietta J.A., Davis M.M., Cohen A.D., Weber K.L., Lancaster E., Mangan P.A., Kulikovskaya I., Gupta M., Chen F. (2020). CRISPR-engineered T cells in patients with refractory cancer. Science.

[45] Locke F.L., Munoz J.L., Tees M.T., Lekakis L.J., de Vos S., Nath R., Stevens D.A., Malik S.A., Shouse G.P., Hamadani M. (2025). Allogeneic Chimeric Antigen Receptor T-Cell Products Cemacabtagene Ansegedleucel/ALLO-501 in Relapsed/Refractory Large B-Cell Lymphoma: Phase I Experience From the ALPHA2/ALPHA Clinical Studies. J. Clin. Oncol..

[46] Jain N., Chevallier P., Liu H., Schiller G.J., Méar J.-B., DeAngelo D.J., Curran K.J., Grupp S., Baruchel A., Balsat M. (2023). Updated Results of the Phase I BALLI-01 Trial of UCART22 Process 2 (P2), an Anti-CD22 Allogeneic CAR-T Cell Product Manufactured By Cellectis Biologics, in Patients with Relapsed or Refractory (R/R) CD22+ B-Cell Acute Lymphoblastic Leukemia (B-ALL). Blood.

[47] Bonini C., Chapuis A.G., Hudecek M., Guedan S., Magnani C., Qasim W. (2023). Genome Editing in Engineered T Cells for Cancer Immunotherapy. Hum. Gene Ther..

[48] Qi C., Gong J., Li J., Liu D., Qin Y., Ge S., Zhang M., Peng Z., Zhou J., Cao Y. (2022). Claudin18.2-specific CAR T cells in gastrointestinal cancers: Phase 1 trial interim results. Nat. Med..

[49] Schober K., Muller T.R., Gokmen F., Grassmann S., Effenberger M., Poltorak M., Stemberger C., Schumann K., Roth T.L., Marson A. (2019). Orthotopic replacement of T-cell receptor alpha- and beta-chains with preservation of near-physiological T-cell function. Nat. Biomed. Eng..

[50] Malki M.M.A., Keyzner A., Popat U.R., Chen Y.-B., Suh H.C., Jain D.T., Solh M.M., Snow A., Pineiro L., Gill S.I. (2025). TSC-100 and TSC-101 Demonstrate the Potential to Reduce Relapse Rates and Increase Relapse-Free Survival in Patients with AML, ALL, or MDS Undergoing Allogeneic Hematopoietic Cell Transplantation (HCT) with Reduced Intensity Conditioning (RIC): Preliminary Results from the Phase 1 Alloha Trial (NCT05473910). Transplant. Cell. Ther..

[51] Kim J.Y., Lee H.-M. (2025). Literature Review on the Development Trends of Gene-Modified Cell Therapies: Focusing on Clinical Studies of TCR-T Cell Therapy. Korean J. Clin. Pharm..

[52] Locke F.L., Munoz J.L., Tees M.T., Lekakis L.J., Eradat H.A., de Vos S., Nath R., Stevens D.A., Malik S.A., Shouse G.P. (2023). ALLO-647 for Lymphodepletion in the Allogeneic CAR T Setting: Safety Experience with ALLO-501/501A in Patients (Pts) with Relapsed/Refractory (r/r) Large B-Cell and Follicular Lymphomas. Blood.

[53] Linette G.P., Stadtmauer E.A., Maus M.V., Rapoport A.P., Levine B.L., Emery L., Litzky L., Bagg A., Carreno B.M., Cimino P.J. (2013). Cardiovascular toxicity and titin cross-reactivity of affinity-enhanced T cells in myeloma and melanoma. Blood.

[54] Eyquem J., Mansilla-Soto J., Giavridis T., van der Stegen S.J., Hamieh M., Cunanan K.M., Odak A., Gonen M., Sadelain M. (2017). Targeting a CAR to the TRAC locus with CRISPR/Cas9 enhances tumour rejection. Nature.

[55] Zheng X., Tian Z. (2025). Engineering TCR-preserved allogeneic CAR-T cells: Leveraging cancer immune evasion principles to establish T-cell tolerance. Cell. Mol. Immunol..

[56] Vantourout P., Hayday A. (2013). Six-of-the-best: Unique contributions of gammadelta T cells to immunology. Nat. Rev. Immunol..

[57] Bank I. (2026). γδ T Cells in Autoinflammatory Diseases. Cells.

[58] Rigau M., Ostrouska S., Fulford T.S., Johnson D.N., Woods K., Ruan Z., McWilliam H.E.G., Hudson C., Tutuka C., Wheatley A.K. (2020). Butyrophilin 2A1 is essential for phosphoantigen reactivity by γδ T cells. Science.

[59] Sebestyen Z., Prinz I., Déchanet-Merville J., Silva-Santos B., Kuball J. (2020). Translating gammadelta (γδ) T cells and their receptors into cancer cell therapies. Nat. Rev. Drug Discov..

[60] Vydra J., Cosimo E., Lesný P., Wanless R.S., Anderson J., Clark A.G., Scott A., Nicholson E.K., Leek M. (2023). A Phase I Trial of Allogeneic γδ T Lymphocytes From Haploidentical Donors in Patients With Refractory or Relapsed Acute Myeloid Leukemia. Clin. Lymphoma Myeloma Leuk..

[61] Li Y.R., Zhu Y., Chen Y., Yang L. (2025). The clinical landscape of CAR-engineered unconventional T cells. Trends Cancer.

[62] Fang X., Yan S., He L., Deng C. (2025). CAR-γδ T cells: A new paradigm of programmable innate immune sentinels and their systemic applications in cancer and beyond. Front. Immunol..

[63] Mukhametshin S.A., Gilyazova E.M., Davletshin D.R., Ganeeva I.A., Zmievskaya E.A., Chasov V.V., Petukhov A.V., Valiullina A.K., Spada S., Bulatov E.R. (2025). Allogeneic NKG2D CAR-T Cell Therapy: A Promising Approach for Treating Solid Tumors. Biomedicines.

[64] Liu C., Li J., Liu D. (2026). Allogeneic B7-H3-Targeted CAR Vδ1T-cell Therapy in Advanced Solid Tumors: A Phase I Study. Clin. Cancer Res..

[65] Nishimoto K.P., Lamture G., Chanthery Y., Teague A.G., Verma Y., Au M., Smith-Boeck M., Salum M., Murthy P., Gundurao S.R.Y. (2025). ADI-270: An armored allogeneic gamma delta T cell therapy designed to target CD70-expressing solid and hematologic malignancies. J. Immunother. Cancer.

[66] Yazdanifar M., Barbarito G., Bertaina A., Airoldi I. (2020). γδ T Cells: The Ideal Tools for Cancer Immunotherapy. Cells.

[67] Deseke M., Trinz I. (2020). Ligand recognition by the γδ TCR and discrimination between homeostasis and stress conditions. Cell Mol. Immunol..

[68] Jhita N., Raikar S.S. (2022). Allogeneic gamma delta T cells as adoptive cellular therapy for hematologic malignancies. Explor. Immunol..

[69] Cieslak S.G., Shahbazi R. (2025). Gamma delta T cells and their immunotherapeutic potential in cancer. Biomark. Res..

[70] Bendelac A., Savage P.B., Teyton L. (2007). The biology of NKT cells. Annu. Rev. Immunol..

[71] Ramos C.A., Courtney A.N., Lulla P.D., Hill L.C., Kamble R.T., Carrum G., Wang T., Pierro E.J.D., Brenner M.K., Heslop H.E. (2024). Off-the-Shelf CD19-Specific CAR-NKT Cells in Patients with Relapsed or Refractory B-Cell Malignancies. Transplant. Cell. Ther. Off. Publ. Am. Soc. Transplant. Cell. Ther..

[72] Niedzielska M., Chalmers A., Popis M.C., Altman-Sharoni E., Addis S., Beulen R., Rudqvist N.P., Chantzoura E., Purbhoo M.A., Chand D. (2025). CAR-iNKT cells: Redefining the frontiers of cellular immunotherapy. Front. Immunol..

[73] Iinuma T., Kurokawa T., Aoki T., Onodera A., Fukazawa T., Yamada D., Kitahara G., Okoshi M., Yamaguchi M., Okura H. (2025). Allogeneic iPSC-derived iNKT cells in recurrent head and neck cancer: A phase 1 trial. Nat. Commun..

[74] Legoux F., Salou M., Lantz O. (2020). MAIT Cell Development and Functions: The Microbial Connection. Immunity.

[75] Dogan M., Karhan E., Kozhaya L., Placek L., Chen X., Yigit M., Unutmaz D. (2022). Engineering Human MAIT Cells with Chimeric Antigen Receptors for Cancer Immunotherapy. J. Immunol..

[76] Li Y.R., Brown J., Yu Y., Lee D., Zhou K., Dunn Z.S., Hon R., Wilson M., Kramer A., Zhu Y. (2022). Targeting Immunosuppressive Tumor-Associated Macrophages Using Innate T Cells for Enhanced Antitumor Reactivity. Cancers.

[77] Toubal A., Nel I., Lotersztajn S., Lehuen A. (2019). Mucosal-associated invariant T cells and disease. Nat. Rev. Immunol..

[78] Li Y.R., Zhou K., Wilson M., Kramer A., Zhu Y., Dawson N., Yang L. (2023). Mucosal-associated invariant T cells for cancer immunotherapy. Mol. Ther..

[79] Themeli M., Kloss C.C., Ciriello G., Fedorov V.D., Perna F., Gonen M., Sadelain M. (2013). Generation of tumor-targeted human T lymphocytes from induced pluripotent stem cells for cancer therapy. Nat. Biotechnol..

[80] Deuse T., Hu X., Agbor-Enoh S., Jang M.K., Alawi M., Saygi C., Gravina A., Tediashvili G., Nguyen V.Q., Liu Y. (2021). The SIRPalpha-CD47 immune checkpoint in NK cells. J. Exp. Med..

[81] Poirot L., Philip B., Schiffer-Mannioui C., Le Clerre D., Chion-Sotinel I., Derniame S., Potrel P., Bas C., Lemaire L., Galetto R. (2015). Multiplex Genome-Edited T-cell Manufacturing Platform for “Off-the-Shelf” Adoptive T-cell Immunotherapies. Cancer Res..

[82] Ren J., Zhang X., Liu X., Fang C., Jiang S., June C.H., Zhao Y. (2017). A versatile system for rapid multiplex genome-edited CAR T cell generation. Oncotarget.

[83] Hawkins R.E., D’Souza R.R., Klampatsa A. (2021). Armored CAR T-cells: The next chapter in T-cell cancer immunotherapy. Biologics.

[84] McGowan E., Lin Q., Ma G., Yin H., Chen S., Lin Y. (2020). PD-1 disrupted CAR-T cells in the treatment of solid tumors; promises and challenges. Biomed. Pharmacother..

[85] Dimitri A., Herbst F., Fraietta J. (2022). Engineering the next-generation of CAR T-cells with CRISPR-Cas9 gene editing. Mol. Cancer.

[86] Roth T.L., Puig-Saus C., Yu R., Shifrut E., Carnevale J., Li P.J., Hiatt J., Saco J., Krystofinski P., Li H. (2018). Reprogramming human T cell function and specificity with non-viral genome targeting. Nature.

[87] Gornalusse G.G., Hirata R.K., Funk S.E., Riolobos L., Lopes V.S., Manske G., Prunkard D., Colunga A.G., Hanafi L.A., Clegg D.O. (2017). HLA-E-expressing pluripotent stem cells escape allogeneic responses and lysis by NK cells. Nat. Biotechnol..

[88] Deuse T., Hu X., Gravina A., Wang D., Tediashvili G., De C., Thayer W.O., Wahl A., Garcia J.V., Reichenspurner H. (2019). Hypoimmunogenic derivatives of induced pluripotent stem cells evade immune rejection in fully immunocompetent allogeneic recipients. Nat. Biotechnol..

[89] Hurton L.V., Singh H., Najjar A.M., Switzer K.C., Mi T., Maiti S., Olivares S., Rabinovich B., Huls H., Forget M.A. (2016). Tethered IL-15 augments antitumor activity and promotes a stem-cell memory subset in tumor-specific T cells. Proc. Natl. Acad. Sci. USA.

[90] Xu Y., Zhang M., Ramos C.A., Durett A., Liu E., Dakhova O., Liu H., Creighton C.J., Gee A.P., Heslop H.E. (2014). Closely related T-memory stem cells correlate with in vivo expansion of CAR.CD19-T cells and are preserved by IL-7 and IL-15. Blood.

[91] Xu X., Huang W., Heczey A., Liu D., Guo L., Wood M., Jin J., Courtney A.N., Liu B., Di Pierro E.J. (2019). NKT Cells Coexpressing a GD2-Specific Chimeric Antigen Receptor and IL15 Show Enhanced In Vivo Persistence and Antitumor Activity against Neuroblastoma. Clin. Cancer Res..

[92] Gattinoni L., Lugli E., Ji Y., Pos Z., Paulos C.M., Quigley M.F., Almeida J.R., Gostick E., Yu Z., Carpenito C. (2011). A human memory T cell subset with stem cell-like properties. Nat. Med..

[93] Rupp L.J., Schumann K., Roybal K.T., Gate R.E., Ye C.J., Lim W.A., Marson A. (2017). CRISPR/Cas9-mediated PD-1 disruption enhances anti-tumor efficacy of human chimeric antigen receptor T cells. Sci. Rep..

[94] Tang N., Cheng C., Zhang X., Qiao M., Li N., Mu W., Wei X.F., Han W., Wang H. (2020). TGF-β inhibition via CRISPR promotes the long-term efficacy of CAR T cells against solid tumors. JCI Insight.

[95] Kloss C.C., Lee J., Zhang A., Chen F., Melenhorst J.J., Lacey S.F., Maus M.V., Fraietta J.A., Zhao Y., June C.H. (2018). Dominant-Negative TGF-β Receptor Enhances PSMA-Targeted Human CAR T Cell Proliferation And Augments Prostate Cancer Eradication. Mol. Ther..

[96] Liu X., Ranganathan R., Jiang S., Fang C., Sun J., Kim S., Newick K., Lo A., June C.H., Zhao Y. (2016). A Chimeric Switch-Receptor Targeting PD1 Augments the Efficacy of Second-Generation CAR T Cells in Advanced Solid Tumors. Cancer Res..

[97] Craddock J.A., Lu A., Bear A., Pule M., Brenner M.K., Rooney C.M., Foster A.E. (2010). Enhanced tumor trafficking of GD2 chimeric antigen receptor T cells by expression of the chemokine receptor CCR2b. J. Immunother..

[98] Di Stasi A., Tey S.K., Dotti G., Fujita Y., Kennedy-Nasser A., Martinez C., Straathof K., Liu E., Durett A.G., Grilley B. (2011). Inducible apoptosis as a safety switch for adoptive cell therapy. N. Engl. J. Med..

[99] Roybal K.T., Rupp L.J., Morsut L., Walker W.J., McNally K.A., Park J.S., Lim W.A. (2016). Precision Tumor Recognition by T Cells With Combinatorial Antigen-Sensing Circuits. Cell.

[100] Lu L., Xie M., Yang B., Zhao W.B., Cao J. (2024). Enhancing the safety of CAR-T cell therapy: Synthetic genetic switch for spatiotemporal control. Sci. Adv..

[101] Russell G.C., Hamzaoui Y., Rho D., Sutrave G., Choi J.S., Missan D.S., Reckard G.A., Gustafson M.P., Kim G.B. (2024). Synthetic biology approaches for enhancing safety and specificity of CAR-T cell therapies for solid cancers. Cytotherapy.

[102] Fraietta J.A., Lacey S.F., Orlando E.J., Pruteanu-Malinici I., Gohil M., Lundh S., Boesteanu A.C., Wang Y., O’Connor R.S., Hwang W.T. (2018). Determinants of response and resistance to CD19 chimeric antigen receptor (CAR) T cell therapy of chronic lymphocytic leukemia. Nat. Med..

[103] Cornu T.I., Mussolino C., Cathomen T. (2017). Refining strategies to translate genome editing to the clinic. Nat. Med..

[104] Kim J.Y., Oh K.-S., Lee H.-M. (2024). A Comparative Study of International Regulatory Framework for Anti-Cancer Immune Cell Therapies. J. Korean Soc. Health-Syst. Pharm..

[105] Roddie C., O’Reilly M., Dias Alves Pinto J., Vispute K., Lowdell M. (2019). Manufacturing chimeric antigen receptor T cells: Issues and challenges. Cytotherapy.

